# Seasonal, Monthly, and Holiday Patterns in Self-Monitored Step Counts Among Individuals with Prediabetes and Type 2 Diabetes: A Two-Year Analysis

**DOI:** 10.3390/ijerph23010053

**Published:** 2025-12-31

**Authors:** Yohannes Woldamanuel, Philip von Rosen, Patrick Bergman, Unn-Britt Johansson, Maria Hagströmer, Jenny Rossen

**Affiliations:** 1 Theme Inflammation and Aging, Karolinska University Hospital, 14186 Huddinge, Sweden; yohannes.woldamanuel@regionstockholm.se; 2 Department of Neurobiology, Care Sciences and Society, Karolinska Institutet, 14183 Huddinge, Sweden; philip.von.rosen@ki.se (P.v.R.); maria.hagstromer@ki.se (M.H.); 3 Department of Medicine and Optometry, eHealth Institute, Linnaeus University, 39182 Kalmar, Sweden; patrick.bergman@lnu.se; 4 Department of Health Promoting Science, Sophiahemmet University, 11486 Stockholm, Sweden; unn-britt.johansson@shh.se; 5 Academic Primary Care Center, 11365 Stockholm, Sweden

**Keywords:** holiday, monthly patterns, physical activity, prediabetes, seasonal variation, step counts, type 2 diabetes

## Abstract

Background: There is a limited amount of evidence concerning the association between seasonal variation and the level of physical activity in individuals with chronic disease. This longitudinal observational study aimed to explore seasonal, monthly, and holiday variations in self-monitored step counts over two years among adults with prediabetes and type 2 diabetes in Sweden. Methods: Participants were recruited at primary care centers from 2013 to 2018 to take part in a physical activity intervention. Inclusion criteria included the following: an age of 40–80 years, having prediabetes or type 2 diabetes (≥1 year), and the ability to communicate in Swedish. Individuals with recent myocardial infarction, impaired renal function, diabetic ulcers, a limited capacity for physical activity, insulin onset (<6 months), recurrent or severe hypoglycemia, a high baseline for physical activity, or no internet access were excluded. In total, 120 participants wore step counters and recorded daily steps for two years. Linear mixed models adjusted for sex, age, and body mass index were applied. Results: Mean (95% CI) step counts were statistically significantly higher in summer (7825 [7762, 7889]) and spring (7805 [7757, 7853]) compared to winter (7098 [7052, 7145]) and fall (7422 [7349, 7490]). Highest step counts were registered in May (7993 [7904, 8071]), followed by June (7968 [7895, 8063]), and the lowest in January (6944 [6856, 7034]) and November (7208 [7113, 7289]). Step counts during holiday periods were statistically significantly lower than non-holiday periods across all seasons. Conclusion: Self-monitored daily steps varied over the seasons in this sample of individuals with prediabetes and type 2 diabetes. Declined physical activity levels in months with unfavorable weather conditions require attention in consultations and research.

## 1. Introduction

Physical activity on a regular basis is critical in the self-management of type 2 diabetes to manage blood glucose control and prevent disease progression [1]. Healthcare providers are therefore encouraged to give support for physical activity to individuals with prediabetes or type 2 diabetes [2]. The physical functioning of adults with chronic diseases, such as type 2 diabetes, can be significantly impacted by both the progression of the disease and the natural aging process [3]. These limitations often result in decreased engagement in physical activity, making individuals with chronic conditions more vulnerable to being less active than the general population [4]. One other significant factor affecting the physical activity levels of adults with chronic conditions is the physical environment, which includes social, built, and natural environments [5]. To create genuinely physical activity-friendly environments and support, it is imperative to consider aspects of seasonal variations, alongside built and social environments [6,7].

Seasonal variations have been shown to influence physical activity and sedentary behavior in general adult populations, showing higher levels of physical activity during summer compared to other seasons, especially winter [8,9,10]. Adverse weather conditions have been reported to influence the level of physical activity in middle-aged adults and are possibly an explanation for seasonal variations [10,11]. In countries with considerable variation in latitude, such as Canada, Norway, and Sweden, regional differences may emerge, and research targeting specific geographic areas has been suggested [9,12]. Holidays associated with cultural, religious, and festival periods may also influence physical activity levels, but their influence has been investigated to a limited extent. A review examining 24 h movement behaviors demonstrated a significant reduction in physical activity levels during Ramadan, accompanied by an increase in sedentary behavior during this time [13]. A prospective study showed decreased light-intensity physical activity among adults during the Christmas and New Year period compared to other times of the year [14].

Earlier studies have focused on more extreme seasonal and weather fluctuations in general populations [8,9,10,11,12,15]. To our knowledge, there is a limited amount of evidence considering the nature and magnitude of the association between seasonal variation and the level of physical activity in individuals with chronic disease [9]. In a recent study including adults with prediabetes and type 2 diabetes, the day-to-day association between weather variables (temperature, sunlight hours, and precipitation) and step counts was examined over a two-year period. It was found that weather accounted for only 10% of the variation in participants’ daily step counts. Conversely, individual factors such as body mass index, age, and sex explained more than 38% of the variation in daily step counts [16]. It is unknown how annual patterns, including seasons, mild winter and summer climates, and holiday periods, impact physical activity in the population with type 2 diabetes [14]. Identifying seasonal variations in physical activity and high-risk periods for behavior change can inform targeted interventions and tailored health messaging [9,10,14].

The aim of this study was to explore seasonal, monthly, and holiday-related variations in self-monitored step counts over a two-year period among adults with prediabetes and type 2 diabetes in Sweden.

## 2. Materials and Methods

This longitudinal observational study utilizes data on self-monitored daily steps from the two intervention arms of the Sophia Step Study, a randomized controlled trial evaluating a physical activity intervention. A detailed description of the study has been published elsewhere [17]. The Stockholm Regional Ethical Review Board approved the study (Dnr. 2012/1570-31/3 and 2015 2075-32), and the study was registered at ClinicalTrials.gov, NCT02374788, on 28 January 2015.

### 2.1. Participants

Participants were recruited over eight rounds from two urban primary healthcare centers in Stockholm and one rural primary healthcare center in southern Sweden. The Sophia Step Study began in 2013, and data collection was completed in 2020. A total of 385 patients were invited, and a general practitioner assessed each participant’s eligibility according to the inclusion and exclusion criteria outlined in Table 1.

Prior to randomization, 188 participants were deemed eligible to participate in the study and were allocated into three arms. One arm consisted of 64 participants assigned to a multi-component intervention, for which the intervention included individual and group counseling with a diabetes specialist nurse and the provision of a step counter for self-monitoring of daily steps. A second arm, assigned to a single-component intervention, comprised 56 participants who were provided with a step counter to self-monitor their daily steps. The third arm served as the control group, with 65 participants receiving standard care. For the current study, aiming to explore seasonal, monthly, and holiday-related variations in self-monitored step counts, we focused exclusively on the two intervention groups.

### 2.2. Data Collection

Each participant was given a step counter (Yamax Digwalker SW200, Yamax Corporation, Tokyo, Japan) and a paper diary to log their daily steps, which they then recorded onto a website for two years. The participants were informed to translate non-ambulant activities (e.g., swimming or cycling) into steps, with 30 min of such activities corresponding to 3500 steps. A text message was sent by the research team every month to participants who had not registered steps during the past four weeks as a gentle reminder and to ask if issues had occurred with login details to the website or the pedometer. Forgotten passwords were reset, and malfunctioning or lost step counters were replaced. During the data cleaning process, some typographical errors were identified in the self-reported step counts. To address this, the data was examined for outliers, and it was determined that any values below 200 or above 40,000 steps per day were unrealistic, thereby treating these entries as missing data.

Data from 2013 to 2019 for seasons, months, and holidays were derived using dates. Seasons were divided into winter, spring, summer, and autumn based on calendar months, and all holidays within each season were grouped together.

### 2.3. Data Analysis

A linear mixed model was used for data analysis. It is an appropriate analysis method given the large number of repeated observations for each participant [19]. However, to minimize the influence of unexpected factors that could distort the associations, the data was treated using a robust linear mixed model approach. Robust linear models can mitigate the impact of very high or low values by assigning weights to the data, thereby reducing the influence of outliers [20]. We modelled the association between seasons, months, and holidays with step counts in separate models. Given that the residuals were not normally distributed, we performed a Box–Cox transformation, which indicated that a square root transformation of the dependent variable was suitable to satisfy the assumption of normally distributed residuals [21]. The *p*-values from the robust linear mixed model were calculated using the Satterthwaite method [22]. Adjustments were made for sex, age, and body mass index, and statistical significance was set at *p* < 0.05. Analyses were conducted in R version 4.3.1 (R core Team, 2003, The R Foundation for Statistical Computing, Vienna, Austria).

## 3. Results

Of the total 120 participants included, 42% (n = 50) were women, with a mean age of 64.6 years (SD = 7.0), 82% (n = 98) were diagnosed with type 2 diabetes, with a mean disease duration of 8.7 years (SD = 6.3), the mean body mass index was 29.1 kg/m^2^ (SD = 4.3), 44% (n = 53) had higher education, and 33% (n = 40) were classified as having low income.

**Figure 1:**
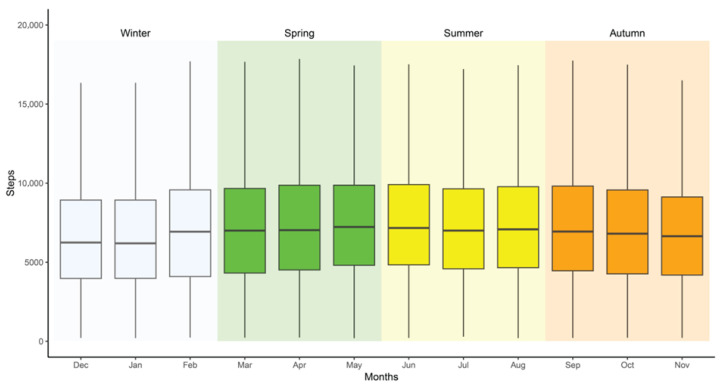
Mean step count by season and month for individuals with prediabetes and type 2 diabetes.

As illustrated in Figure 1, mean daily step counts were statistically significantly higher during summer (7825 steps, 95% CI [7762–7889]) and spring (7805 steps, 95% CI [7757–7852]) compared to winter (7098 steps, 95% CI [7052–7145]) and fall (7422 steps, 95% CI [7349–7490]). Among individual months, May recorded the highest mean step count (7993 steps, 95% CI [7904–8071]), closely followed by June (7968 steps, 95% CI [7895–8063]), whereas the lowest means were observed in January (6944 steps, 95% CI [6856–7034]) and November (7208 steps, 95% CI [7113–7289]).

Table 2 presents the mean step counts during holiday periods, and Table 3 shows the differences between seasonal means and holidays within each season. Step counts were statistically significantly lower during holidays compared with non-holidays in winter (*p* < 0.001), spring (*p* < 0.001), and summer (*p* = 0.003). Among the holidays, Ascension Day had the highest step counts, while Christmas and New Year’s Eve recorded the lowest.

## 4. Discussion

This study explored seasonal-, monthly-, and holiday-related variations in self-monitored step counts over two years among adults with prediabetes and type 2 diabetes living in southern Sweden. The results indicate noticeable patterns influenced by season, month, and holidays. Step counts were higher in spring and summer and dropped significantly in winter, suggesting that warmer temperatures and longer daylight hours encourage more physical activity. May, June, and July were identified as the most favorable months. Moreover, the data indicates an evident decline in the daily steps during public holidays, when compared to the activity levels observed during non-holiday days for the respective seasons.

The findings are consistent with previous research demonstrating a significant influence of seasonal variations on physical activity behavior in general populations [9,10,13], as well as among older adults across diverse environmental contexts [23,24]. When examining the associations of the same step data with daily weather conditions (temperature, daylight hours, and precipitation), a trend was observed showing increased daily steps on days with higher temperatures, longer daylight hours, and minimal precipitation. However, this trend was not statistically significant [16]. This suggests that, in this Swedish sample of individuals with prediabetes and type 2 diabetes, while weather factors such as temperature and daylight can influence activity levels, the impact of extreme seasonal differences, particularly between summer and winter, may have a more pronounced influence on daily steps. Research studying the influence of weather factors on physical activity has shown that more extreme weather impacts physical activity levels [10,11], but weather and climate aspects need further investigation [9,10]. Other aspects of seasonal changes, rather than daily variations in weather, may influence physical activity levels [13]. During winter, physical activity may also decrease due to cold and flu season, personal barriers, such as low motivation, health concerns, fear of injury, and the overall inconvenience of exercising in cold weather. These seasonal shifts likely result in a cumulative effect that influences the activity patterns more consistently over time, while monthly or weather variations capture short-term fluctuations. Comparing these perspectives will help us understand how physical activity behaviors respond to both long-term shifts and immediate weather changes. This highlights the need to consider both seasonal and weather factors when evaluating and promoting physical activity behaviors [9,10,14].

The holiday-related physical activity patterns observed in the present study are consistent with prior research, which has documented a decrease during Ramadan [13] and the Christmas and New Year period [14]. This is of importance to individuals with type 2 diabetes, as there are indications of seasonal influences on metabolic and vascular functions affected by changes in physical activity levels [25]. The possible health effects, such as increased blood pressure, blood glucose, and blood lipids, from a short-term decline in physical activity during holidays require further investigation.

A profound strength of this study lies in the amount of data utilized, with two years of daily step counts for each individual. As data collection took place from 2013 to 2020, the study covers seven years, with variations in weather and trends in society. Additionally, holidays were analyzed as a factor that might influence engagement in physical activity, an aspect that has not yet been explored among people with prediabetes and type 2 diabetes.

A limitation of the study is the use of self-reported step counts. The accuracy of step counts recorded by the pedometer may be affected by the body composition of the participants and device placement, which can lead to underestimation of physical activity levels [26]. Further, self-reported steps introduce the potential for social desirability and recall bias. Participants may have overestimated their activity levels to present themselves in a more favorable light or forgotten to notice the step counts and registered steps in hindsight, based on their memory or based on usual days. However, the long data collection period reduces the influence of potential misreporting on the association with seasonal variations. Moreover, the data was checked for outliers and unusual amounts of steps, and it was treated using a robust linear mixed model approach; therefore, the influence of outliers was reduced. The study did not differentiate between steps from indoor and outdoor activities, which may have influenced the interpretation of step-count variations.

There are some concerns regarding the representativeness of the study. The findings are most relevant to regions with climates similar to southern Sweden but may not be directly transferable to areas with distinct climate conditions. Moreover, the study is based on a sample of individuals partaking in a physical activity intervention, which included goal-setting, among other behavior change techniques. It is likely that the participants have integrated strategies to maintain physical activity levels over the year [15,27].

Future studies utilizing data from large cohorts with device-measured physical activity, or RCTs with control conditions, should further investigate how seasonal, geographical, cultural, and weather aspects influence activity patterns both in general populations as well as in populations with chronic disease.

## 5. Conclusions

The present study identified seasonal variations in self-reported daily steps among individuals with prediabetes and type 2 diabetes over a two-year period. The findings suggest that in the south of Sweden, certain months—particularly November through January—offer less favorable conditions for step-based activities compared to other months. When evaluating and promoting physical activity behaviors, both seasonal and weather-related factors should be considered.

## Figures and Tables

**Table 1:** Inclusion and exclusion criteria.

Inclusion Criteria	Exclusion Criteria
HbA1c > 39 mmol/mol < 47 mmol/L, fasting blood glucose > 5.6 mmol/L (prediabetes), or diagnosed with type 2 diabetes with a duration of ≥1 year.	Having had a myocardial infarction in the past six months
Age 40–80 years	Serum creatinine > 140 µmol/L
Ability to read, write, and express themselves in the Swedish language.	Diabetic ulcer or risk for ulcer
	Prescribed insulin in the past six months
	Additional disease prohibiting physical activity
	Having experienced repeated hypoglycemia or severe hypoglycemia in the last 12 months
	Being physically active, according to the Stanford Brief Activity Survey [18]
	Having no access to the internet

**Table 2:** Mean daily step count at public holidays among individuals with prediabetes and type 2 diabetes.

Holiday	Steps/Day Mean (sd)
Christmas	5998 (3908)
New Year’s Eve	5916 (3694)
Epiphany	6629 (4067)
Easter	7190 (4175)
May 1st	7282 (4496)
Ascension Day	7955 (4398)
Midsummer Day	7036 (4047)
National Day	7506 (3810)
All Saints’ Day	7032 (4044)

**Table 3:** Mean step count by season, compared with public holidays at each respective season, among adults with prediabetes and type 2 diabetes.

Season/Holidays	Steps/Day Mean (95% CI)	*p*-Value
Winter	7098 (7053–7145)	<0.001
Winter holidays	6185 (6029–6389)	
Spring	7805 (7757–7853)	<0.001
Spring holidays	7455 (7281–7656)	
Summer	7825 (7762–7889)	0.003
Summar holidays	7292 (6983–7676)	
Fall	7422 (7349–7490)	0.195
Fall holidays	7107 (6549–7492)	

*p*-values were derived from robust linear mixed models. Adjustments were made for sex, age, and body mass index; statistical significance was set at *p* < 0.005.

## Data Availability

The raw data supporting the conclusions of this article will be made available by the authors on request.
